# Detecting, reporting, and analysis of priority diseases for routine public health surveillance in Liberia

**DOI:** 10.11604/pamj.supp.2017.27.1.12561

**Published:** 2017-05-28

**Authors:** Joseph Asamoah Frimpong, Maame Pokuah Amo-Addae, Peter Adebayo Adewuyi, Casey Daniel Hall, Meeyoung Mattie Park, Thomas Knue Nagbe

**Affiliations:** 1 Liberia Field Epidemiology Training Program, Monrovia, Liberia; 2 Rollins School of Public Health, Emory University, Atlanta, USA; 3 Ministry of Health, Monrovia, Liberia

**Keywords:** Public Health, epidemiology, surveillance, data quality audit, data

## Abstract

Public health officials depend on timely, complete, and accurate surveillance data for decision making. The quality of data generated from surveillance is highly dependent on external and internal factors which may either impede or enhance surveillance activities. One way of identifying challenges affecting the quality of data generated is to conduct a data quality audit. This case study, based on an audit conducted by residents of the Liberia Frontline Field Epidemiology Training Program, was designed to be a classroom simulation of a data quality audit in a health facility. It is suited to enforce theoretical lectures in surveillance data quality and auditing. The target group is public health trainees, who should be able to complete this exercise in approximately 2 hours and 30 minutes.

## How to use this case study

**General instructions:** a class of up to 20 trainees is ideal for a training sessions using this case study. The instructor facilitating the session should direct a participant to read a paragraph out loud, going around the room to give each participant a chance to read. Based on the type of question, the instructor may decide to divide the class into small groups for exercises, randomly identify a trainee to respond to the question, or engage the class in a group discussion of the answer. The aim of the interaction is to allow participants to learn from each other and not just from the instructor. Specific instructor’s notes are included with each question in the instructor’s version of this case study.

**Audience:** residents in Frontline Field Epidemiology Training Programs (FETP-Frontline), Field Epidemiology and Laboratory Training Programs (FELTPs), and others who are interested in this topic.

**Prerequisites:** for this case study, trainees should have received lectures on data quality, data quality auditing, and SWOT analysis.

**Materials needed:** flipchart or white board with markers

**Level of training and associated public health activity:** Novice – Data Quality Auditing

**Time required:** approximately 2-3 hours

**Language:** English

## Case study material

- Download the case study student guide (PDF - 1.50 MB)
- Request the case study facilitator guide
